# Interfacility transfers in a non-trauma system setting: an assessment of the Greek reality

**DOI:** 10.1186/1757-7241-18-14

**Published:** 2010-03-16

**Authors:** Stylianos Katsaragakis, Panagiotis G Drimousis, Eleftheria S Kleidi, Kostas Toutouzas, Eleftherios Lapidakis, Georgios Papadakis, Kritolaos Daskalakis, Andreas Larentzakis, Maria E Theodoraki, Dimitrios Theodorou

**Affiliations:** 1 First Department of Propaedeutic Surgery, Surgical Intensive Care Unit, Hippocration General Hospital, Athens, Greece; 2 First Surgical Department, General Hospital KAT, Athens, Greece; 3 Second Surgical Department, General Hospital KAT, Athens, Greece; 4 Surgical Department, General Hospital of Rethymno, Rethymno, Greece; 5 Biostatistician, First Propaedeutic Surgical Clinic, Hippocration General Hospital, Athens Medical School, Greece

## Abstract

**Background:**

Quality assessment of any trauma system involves the evaluation of the transferring patterns. This study aims to assess interfacility transfers in the absence of a formal trauma system setting and to estimate the benefits from implementing a more organized structure.

**Methods:**

The 'Report of the Epidemiology and Management of Trauma in Greece' is a one year project of trauma patient reporting throughout the country. It provided data concerning the patterns of interfacility transfers. We compared the transferred patient group to the non transferred patient group. Information reviewed included patient and injury characteristics, need for an operation, Intensive Care Unit (ICU) admittance and mortality. Analysis employed descriptive statistics and Chi-square test. Interfacility transfers were then assessed according to each health care facility's availability of five requirements; Computed Tomography scanner, ICU, neurosurgeon, orthopedic and vascular surgeon.

**Results:**

Data on 8,524 patients were analyzed; 86.3% were treated at the same facility, whereas 13.7% were transferred. Transferred patients tended to be younger, male, and more severely injured than non transferred patients. Moreover, they were admitted to ICU more often, had a higher mortality rate but were less operated on compared to non transferred patients. The 34.3% of transfers was from facilities with none of the five requirements, whereas the 12.4% was from those with one requirement. Low level facilities, with up to three requirements transferred 43.2% of their transfer volume to units of equal resources.

**Conclusion:**

Trauma management in Greece results in a high number of transfers. Patients are frequently transferred between low level facilities. Better coordination could lead to improved outcomes and less cost.

## Background

The goal of a trauma system is to consistently get the right patient to the right type of hospital in the right amount of time. This requires an organized and coordinated process [1]. Trauma systems are designed to centralize resources and experience, to assure appropriate access to definite care and to maximize efficiency [2,3]. Although trauma systems have reduced trauma mortality in the USA [4-7], most European countries including Greece do not have formal systems.

Interfacility transfers are resource intensive, particularly in rural regions where patients may require transport over hundreds of miles, and transport time may delay definite treatment [8]. Transfers from rural to urban areas are often required for specialist expertise and/or more sophisticated treatment options [9]. Quality assessment for any established or proposed trauma system requires the evaluation of transport patterns.

The National Health System in Greece is government funded and has three levels of health care facilities. Primary health care units are general practitioner based outpatient facilities. They are mostly implemented in remote areas to address the need for emergency care. Secondary health care units are general hospitals. In most cases, these structures are situated in the capital of each of the 50 prefectures. There is considerable diversity among these facilities. Those that are situated in major cities may be quite sophisticated whereas those in remote, rural prefectures usually have limited resources and personnel. The third degree of health care (tertiary units) consists of specialized centers and university hospitals which are primarily in major cities. Secondary health care units may function like a Level II, III or IV trauma center, whereas a tertiary unit has capabilities similar to a Level I or II trauma center. Emergency incidents in our country are referred to the regional primary or secondary health care facility and if further resources are needed, patients are transferred to a higher level center. There are some private hospitals but these are mostly located in major cities.

In many ways, Greece is highly urbanized and more than 40% of the population lives in the two major cities. However, there are hundreds of habitable islands many with limited access to the mainland.

The National Emergency Center is the only official carrier responsible for patient transportation in Greece. Transfers from islands are performed by ground, air or water. Transfer means personnel are mostly paramedics with varying degrees of emergency care training. There is a coordinating authority in the National Emergency Center but it has radio access only to the ambulances and not to the receiving hospital.

The purpose of the present study is to assess interfacility transfers in a country with discrete needs of transportation and no official trauma system implementation. For this reason data out of a countrywide registry are presented and initially analyzed.

## Methods

### Study Design and Population

The 'Report of the Epidemiology and Management of Trauma in Greece' is a project initiated by the Hellenic Society of Trauma and Emergency Surgery in October 2005. It represents an effort to register and evaluate the epidemiology of trauma in Greece and to critically assess the management of trauma patients in the country. The project comprises prospectively collected data on trauma patients from October 2005 till October 2006.

There are 99 hospitals in Greece but only 80 accept trauma patients. All facilities with surgeons who served as official representatives of the Society were invited to participate in the registry. Thirty two representatives responded positively accounting for 40% of hospitals accepting trauma patients. The participating hospitals provide trauma care for approximately 40% of the country's residents.

All reporting data forms were completed prospectively by the official representative surgeons and mailed to the Board of the Society on a monthly basis. A physician transferred the data to an electronic database for future statistical evaluation. The database was subsequently cross checked for double entries (patients who had been reported by both sending and receiving hospitals) and accuracy by an independent physician. Reports with more than 5% missing data were excluded from the database. Inclusion criteria for the registry were defined as either trauma patients that required admission, or transfer to another unit or arrived dead or died in the Emergency Department. International recommendations for uniform reporting of data following trauma were followed [10,11].

### Setting and data collection

Specific fields in the reporting data form noted whether the trauma patient was transferred in from another health care unit, or transferred out to another hospital. Transferred patients were defined as those patients that arrived at the reporting hospital after being initially evaluated or managed in another health care unit or transported from the reporting unit to another hospital. Non transferred patients were defined as those patients who were definitely treated at the reporting hospital. Injured military personnel and trauma patients with a private health care insurance who requested a transfer to a private hospital were excluded from the analysis. Legislation in Greece requires that all military personnel are transferred to a military hospital after initial evaluation and care irrespective of the medical indications. Database trauma patients that arrived dead directly from the scene of injury or died in the Emergency Department were also excluded from this analysis. The transferred group was then compared to the non transferred group and the variables reviewed included gender, age, Injury Severity Score (ISS), mechanism and cause of injury, need for an operation, need for ICU admittance and mortality.

### Additional data collection

In order to accurately evaluate the actual resources of each facility the data collection form specifically inquired for the availability of an Intensive Care Unit (ICU) and of a Computed Tomography (CT) scanner on a 24 hour basis, and for the presence of a neurosurgeon, an orthopedic surgeon and a vascular surgeon in both the Emergency Department and the hospital. For each of these prerequisites a point was given to each facility. A score of five out of five (5/5) implies that the facility's infrastructure satisfied all five requirements, whereas a score of zero out of five (0/5) suggests that the facility did not have any of the above. Furthermore, facilities were divided into two levels; "low level" facilities met up to three requirements, whereas "high level" facilities had four or five requirements.

The official representatives were asked to provide additional information for each institution, concerning the availability of resources and specific specialties in the receiving and the referring facility.

### Ethics

This sub-study was reviewed and approved by the Hellenic Society of Trauma and Emergency Surgery. The Society is a non governmental entity, authorized under Greek law to design, approve and finance observational studies in relevant scientific fields.

### Data Analysis

Statistical analyses were performed using chi square test for comparison of data. Pearson chi square test was used for parametric variables and Mann-Whitney test for non-parametric ones. Data are represented as mean ± SD (standard deviation), unless otherwise specified.

## Results

After excluding the 11 soldiers, 60 private insurance patients and trauma patients who were dead on arrival, data on 8,524 patients were included in the analysis. Of them, 7,359 (86.3%) arrived directly from the scene and were treated at the same facility, whereas 1,165 (13.7%) were transferred. Of the 1,165 total transfers, 536 (46%) were transferred in to the reporting hospital, 569 (48.8%) were transferred from the reporting hospital to another facility and 60 patients (5.2%) were double transferred; i.e. transferred from health care unit A to B and from B to C, where B is the reporting hospital in the registry. Table 1 shows these data for the total number of transfers reported in this study.

**Table 1:** Distribution of transfers in the total number of patients

PATIENTS		N	% of TP*	% of TRP*
**Transferred**	From another unit	536	6.3	46.0
	To another unit	569	6.7	48.8
	From and to another unit	60	0.7	5.2
	
	Total transferred	1165	13.7	100

**Non Transferred**	Total non transferred	7359	86.3	
	
**TOTAL**	Total patients	8524	100	

* TP, Total Patients; TRP, Transferred Patients

**Figure 1:**
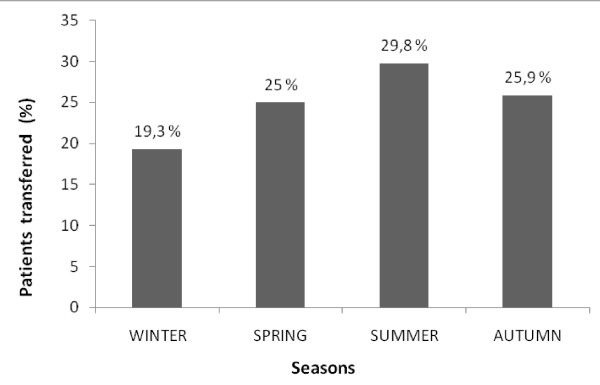
**Seasonal distribution of interfacility transfers**.

Figure 1 shows the seasonal distribution of transfers. There is a substantial increase in transfers during summer and a respective fall during winter.

Table 2 shows the differences in patient and injury characteristics between transferred and non transferred patients. In transferred group there were more male, the mean age was lower than that of the non transferred group and the injury severity score was higher. As for the mechanism of injury that led to a transfer, 27.5% of burned patients were transferred, followed by blunt (13.6%) and penetrating trauma (11.6%). Regarding the cause of injury, 16.9% of patients with industrial injuries were transferred and concomitantly were motor vehicle collisions (16.4%) and falls (10.9%).

**Table 2:** Differences in patient and injury characteristics between Transferred and Non Transferred patients

CHARACTERISTIC		NON TRANSFERRED	TRANSFERRED
**Gender, N (%)**	Female	2409 (89.2)	291 (10.8)
	Male	4950 (85.0)	874 (15.0)

**Age (y)**	(mean ± SD)	45.8 ± 22.2	40.6 ± 23.4

**I.S.S**	(median ± IQR)*	4.0 ± 5.0	9.0 ± 9.0

**Mechanism of injury**	Burn	108 (72.5)	41 (27.5)
**N (%)**	Blunt	6459 (86.4)	1015 (13.6)
	Penetrating	374 (88.4)	49 (11.6)
	Unknown	418 (87.4)	60 (12.6)

**Cause of injury**	Industrial	452 (83.1)	92 (16.9)
**N (%)**	MVC**	3028 (83.6)	596 (16.4)
	Fall	2500 (89.1)	306 (10.9)
	Illegal act	432 (91.9)	38 (8.1)
	Sports	130 (94.2)	8 (5.8)
	Other	233 (82.6)	49 (17.4)
	Unknown	584 (88.5)	76 (11.5)

* Inter quartile range

** MVC, Motor Vehicle Collision

Table 3 shows the frequency of operation and ICU admission, as well as the mortality rates in each group. Transferred patients were less likely to be operated on (19.9% versus 30%) but were admitted to ICU in a higher percentage (2.7% versus 1.3% respectively). Moreover, 3.5% of the transferred patients died, whereas the mortality of non transferred patients was 2%.

**Table 3:** Frequency of operation, ICU* admission and mortality rates in Transferred and Non Transferred patients

		NON TRANSFERRED		TRANSFERRED	
**Operation, N (%)**	No	5153	70.0%	933	80.1%
	Yes	2206	30.0%	232	19.9%

**ICU admission, N (%)**	No	7265	98.7%	1134	97.3%
	Yes	94	1.3%	31	2.7%

**Dead, N (%)**	No	7211	98.0%	1124	96.5%
	Yes	148	2.0%	41	3.5%

* ICU, Intensive Care Unit

**Figure 2:**
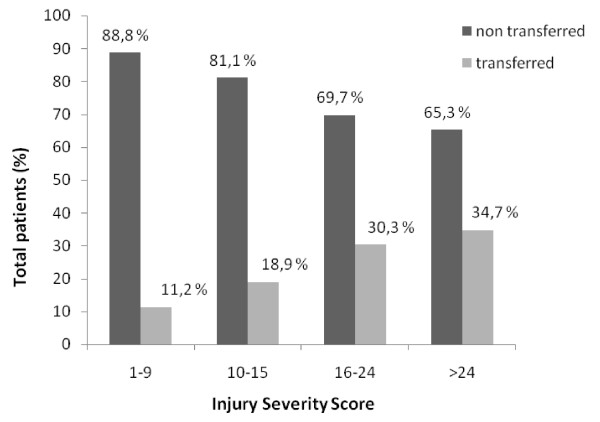
**Comparison of transferred and non transferred patients regarding Injury Severity Score**.

The comparison of transferred and non transferred patients' ISS is shown in Figure 2. The most severely injured patients (ISS >24) were transferred more often (34.7%), whereas 11.2% of patients with minor injuries (ISS 1-9) were transferred.

Table 4 groups the health care facilities in two categories: low level facilities that met up to three requirements and high level facilities that met four or more requirements (ICU, CT scanner, neurosurgeon, orthopedic surgeon and vascular surgeon). Low level facilities transferred 43.2% of those patients that they did transfer to other low level facilities and only 55.4% to a higher level unit. Moreover, 18.4% of the transferred patients went from a high level facility to a lower level facility.

**Table 4:** Distribution of transfers according to the level of the referring and the receiving facility.

		RECEIVING FACILITY						
		
		Low^a^	(%)	High^b^	(%)	Unk^c^	(%)	Total
**REFERRING FACILITY**	**Low^a^**	397	(43.2)	510	(55.4)	13	(1.4)	920
	**High^b^**	34	(18.4)	137	(74.1)	14	(7.6)	185

**TOTAL**		431		647		27		1105^d^

^a ^Low level: up to three out of five requirements (Computed Tomography scanner, Intensive Care Unit, neurosurgeon, orthopedic and vascular surgeon)

^b ^High level: four or more out of five requirements

^c ^Unk, Unknown

^d ^Total transfers after excluding double-transferred patients

## Discussion

Interfacility transfers are an integral part of any health care system particularly one focused on trauma care. Coordinated trauma care requires that an infrastructure exists for the transfer of injured patients [12]. There are numerous prerequisites for optimal outcome: a central coordinating authority, a well equipped ambulatory service, radio-communication between the ambulances and the receiving hospital, predefined arrangements between hospitals in the same geographical area, adequately trained paramedics and strict protocols of action are only few of these [13]. Optimally, trauma patients should go directly from the scene to the right hospital for the injury they sustained. This is not always the nearest facility. Even when the closest facility is appropriate for initial stabilization subsequent transfers should be made as quickly and efficiently as possible. This process is expedited by a systems approach [14,15].

Quality assessment of any trauma system involves the evaluation of the transferring patterns [16]. The geographic diversity and population distribution of Greece renders this evaluation quite challenging. This is made worse by the lack of predefined agreements between institutions; accepted transporting guidelines and the fact that responsibility for transfers belongs to the referring physician and to the National Emergency Center, which typically has limited access to vital information.

The overall transfer rate observed (13.7%) is one of the highest rates reported in studies with similar inclusion criteria [8,15,16]. In our opinion, the main reason for this is that the patients are transferred to the closest rather than to the most suitable institution. Moreover, the prefecture-based infrastructure of the health care system produces a misleading situation; all prefectures have at least one 'secondary' hospital that receives all trauma patients in the prefecture, but all hospitals designated as 'secondary' are not the same. In realistic terms, trauma patients may be transferred from the scene to a facility that is totally unsuitable for their injury, and therefore the necessity for further management will remain, leading to increased transfer rates.

A number of recent studies have reported several reasons for patient transfer. These include gender, age, race, specialist availability and referring facility's characteristics [1-3,17,18]. In our study, transferred patients tended to be younger and male. Transfer rates are relatively high during summer when the majority of the population travels for vacations and when millions of tourists arrive in the country, mostly in the islands where the health care resources are relatively poor.

Injury severity plays an important role in the decision making of a transfer [19]. Transferred patients did have a higher median Injury Severity Score than non transferred ones but most patients had relatively minor injuries (median ISS 9.0 and 4.0 respectively). In addition, transfer rates increased proportionately to ISS categories implying a proper manner of interfacility transfer. However, fifty percent of transferred patients had an ISS lower than 10 which implies over triage or primary referral to an inappropriate center. The latter may be reflected by the fact that referring units with no requirements -as defined in our study- transferred 34.3% of the total transferred patients, whereas units with at least one transferred only 12.4%. Community health centers do serve as a local resource to care for minor injuries, which helps to reduce the burden on the more specialized units and avoid unnecessary transport over long distances but they are incapable of treating more serious injuries. In our geographical setting, where transfer over sea distances can be complex and expensive coordination between primary and specialized centers is essential.

Unlike other studies [1,3,6,17,20], we found that burns were overrepresented among transferred patients. This can be attributed to the fact that there are three centers specialized in treating burn injuries in the whole country and they are situated in the two major cities. Even though the total number of patients transferred for this reason is relatively low, it may still indicate the need of a more extensive distribution of specialized centers throughout the country.

The fact that 43.2% of the transfers from hospitals with 3 or fewer resources actually go to a hospital of the same level implies that the stated resources (ICU beds, CT scanners or operating rooms) may not always be available, Interestingly enough, hospitals with just one out of five requirements (in most cases it is the presence of a CT scanner) are responsible for the 12.4% of the total transfer volume, whereas facilities with 2/5 requirements are responsible for the 17.4% of the total number of transfers. On the other hand, the fact that 34.3% of all transfers are from units with no requirements at all implies that the presence of a single requirement may result in a substantial decrease in the frequency of transfers. This indicates the demand of a more organized distribution of resources.

## Limitations

One of the major drawbacks of this study is that in most cases only one of the facilities involved participated in the registry. Personal communication of the official representatives with the receiving hospitals added some missing data. These mainly involved patient additional injuries and outcome but still there are missing points. There is also data missing about the initial evaluation and management when only the receiving facility reported.

## Conclusions

Our results show that the current system of trauma management in Greece has a high number of transfers and that patients are frequently transferred between two facilities with low resources. A more coordinated system would be more efficient. This would likely result in better outcomes. Since the Greek health care system is publically funded, reducing the number of transfers would probably reduce overall costs as well. This research group from the Hellenic Society of Trauma and Emergency Surgery will assess these changes as they occur.

## List of Abbreviations

CT: Computed Tomography; ICU: Intensive Care Unit; ISS: Injury Severity Score; SD: Standard Deviation.

## Competing interests

The authors declare that they have no competing interests.

## Authors' contributions

SK conceived the study, undertook recruitment of participating centers and chaired the data oversight committee. PGD analyzed the data and critically revised the manuscript. KT designed the trial and was responsible for the quality control of the study. EL undertook recruitment of participating patients and supervised the data collection. KD undertook recruitment of participating patients and supervised the data collection. AL transferred the data in electronic form and drafted the manuscript. GP undertook recruitment of participating patients and supervised the data collection. MET provided statistical advice on study design, analyzed the data and was responsible for the quality control of the study. ESK transferred the data in electronic form, analyzed the data and drafted the manuscript. DT designed the trial, supervised the conduct of the trial and critically revised the manuscript. All authors approved the final version.
